# The Cost-Effectiveness of Adding Helicobacter Pylori Screening to the National Gastric Cancer Screening Program in Korea: Protocol for a Cost-Effectiveness Modeling Study

**DOI:** 10.2196/72228

**Published:** 2025-07-15

**Authors:** Seowoo Bae, Joon Sung Kim, Moon Won Lee, Gwang Ha Kim, Young-Il Kim, Woon Tae Jung, Gwang Ho Baik, Beom Jin Kim, Joongyub Lee, Mina Suh, Jae Gyu Kim

**Affiliations:** 1 National Cancer Control Institute, National Cancer Center Goyang Republic of Korea; 2 Department of Internal Medicine, Incheon St. Mary's Hospital, College of Medicine, The Catholic University of Korea Seoul Republic of Korea; 3 Department of Internal Medicine, Pusan National University School of Medicine and Biomedical Research Institute, Pusan National University Hospital Busan Republic of Korea; 4 Center for Gastric Cancer, Research Institute and Hospital, National Cancer Center Goyang Republic of Korea; 5 Department of Internal Medicine and Institute of Medical Science, College of Medicine, Gyeongsang National University and Hospital Jinju Republic of Korea; 6 Department of Internal Medicine, Hallym University College of Medicine Chuncheon Republic of Korea; 7 Department of Internal Medicine, Chung-Ang University College of Medicine Seoul Republic of Korea; 8 Department of Preventive Medicine, Seoul National University College of Medicine Seoul Republic of Korea

**Keywords:** eradication, gastric cancer, Helicobacter pylori, primary prevention, screening

## Abstract

**Background:**

In Korea, the National Cancer Screening Program (NCSP) was implemented in 1999 and provides biennial endoscopy for adults aged ≥40 years. The NCSP has contributed to the early detection of gastric cancer and reduction of associated mortality in Korea. *Helicobacter pylori* is the main cause of gastric cancer. Screening for and eradication of *H pylori* reduces the incidence and mortality of gastric cancer. Previous studies have reported that screening for *H pylori* is a cost-saving intervention that can significantly decrease gastric cancer burden in areas with a high prevalence of *H pylori* infection. However, no study has examined whether incorporating *H pylori* screening into national endoscopic screening is cost effective.

**Objective:**

This study aims to evaluate the cost-effectiveness of incorporating *H pylori* screening into Korea’s National Gastric Cancer Screening Program.

**Methods:**

We have developed a Markov model to compare two strategies: (1) endoscopy screening every 2 years starting at the age of 40 years (conventional screening), and (2) *H pylori* screening at the age of 40 years followed by continuous endoscopy screening every 2 years. We will also conduct a comparative analysis by varying the age at which the *H pylori* screening is performed. The primary outcome is the incremental cost-utility ratio (ICUR), calculated by dividing the incremental cost by the incremental quality-adjusted life-years (QALYs) between the two strategies. A probabilistic sensitivity analysis will be performed to test the uncertainty of the cost-effectiveness results. A sensitivity analysis will identify the most influential variables for cost-effectiveness.

**Results:**

The primary outcome parameter is the cost-effectiveness of adding *H pylori* testing to the current NCSP, which is expressed as the ICUR. Costs and utilities are discounted at an annual rate of 4.5%. The ICUR threshold is set at KRW 50 million (US $36,719), which is the South Korean gross domestic product per capita. This research has been funded by a Patient-Centered Clinical Research Coordinating Center grant from the Ministry of Health & Welfare, Republic of Korea (grant RS-2024-00398474). This study will analyze and synthesize previously published information and is thus exempt from institutional review board approval. Data collection started in June 2024 and was completed in May 2025. Study results will be published in peer-reviewed journals and presented at national and international conferences throughout 2025.

**Conclusions:**

We will examine whether introducing *H pylori* testing and eradication therapy into the NCSP is a more cost-effective strategy for reducing gastric cancer risk than conventional endoscopy-based screening. Our study also examines the optimal age for *H pylori* screening, as well as the optimal screening frequency.

**International Registered Report Identifier (IRRID):**

DERR1-10.2196/72228

## Introduction

*Helicobacter pylori* infection is associated with peptic ulcers, marginal zone B-cell lymphoma of the mucosa-associated lymphoid tissue type, and gastric cancer [1]. South Korea has the highest global incidence rate of gastric cancer and a high prevalence of *H pylori* [2,3]. The prognosis of cancer is closely related to its stage at the time of the first diagnosis. Therefore, early detection is crucial to avoid death. Endoscopic screening reduces the risk of gastric cancer mortality by 40% [4]. Furthermore, early gastric cancer patients have a high chance of improving their long-term quality of life through treatments such as endoscopic mucosal resection or endoscopic submucosal dissection [5]. Therefore, early detection with an effective screening strategy is crucial in high-incidence countries such as Korea. To prevent this high incidence of serious severity or death, the Korean government implemented the National Cancer Screening Program (NCSP) in 2002 and currently facilitates adults aged ≥40 years to undergo upper gastrointestinal series or endoscopy as primary gastric cancer screening every 2 years [6]. The optimal frequency for gastric cancer screening is still debated, and it is unclear whether tailoring screening intervals according to an individual’s gastric cancer risk may outperform the current NCSP approach of repeating endoscopy every 2 years.

Eradication of *H pylori* is associated with a 52% reduction in gastric cancer incidence and a 38% reduction in mortality [7]. *H pylori* screening and eradication have been reported to be associated with significant cost savings and health impacts for gastric cancer prevention [8]. A recent study reported that nationwide endoscopy screening is not cost-effective and that cost-effectiveness can be improved when *H pylori* eradication is performed only in high-risk groups [9]. For many years, South Korea and Japan have implemented nationwide programs to screen for gastric cancer. A recent study reported that South Korea’s NCSP has apparent benefits for decreasing gastric cancer mortality and upper gastrointestinal disease mortality [10]. This study aims to determine whether adding *H pylori* screening to the current NCSP in South Korea would result in greater benefits in terms of cost compared to endoscopic screening alone.

## Methods

### Model Design

We constructed a Markov model to perform a cost-effectiveness analysis to evaluate the economic benefits of incorporating *H pylori* testing and eradication therapy into the existing endoscopy-based NCSP. Markov models can track long-term progression of diseases with complex progression, such as cancer, so they are widely used in studies to predict long-term follow-up cost-effectiveness for chronic diseases [11].

We referenced a previously published Markov model for the economic evaluation of gastric cancer screening in Korea, modifying it to focus on *H pylori* infection [12]. Therefore, the cancer screening–related data input into the model were based on the cancer screening and registration data in Korea as used in Bae et al [12]. Other epidemiological indicators were reviewed by multiple expert review meetings to use real-world data as much as possible. This study is being conducted with reference to the Consolidated Health Economic Evaluation Reporting Standards (CHEERS) framework (Multimedia Appendix 1).

The two main strategies being compared for cost-effectiveness are (1) a strategy in which individuals undergo endoscopy every 2 years starting at the age of 40 years (conventional screening), and (2) a strategy in which individuals receive both endoscopy and *H pylori* testing with eradication therapy at the age of 40 years followed by continuous endoscopy thereafter. Furthermore, we will conduct a comparative analysis by varying the age at which *H pylori* screening is performed.

**Figure 1:**
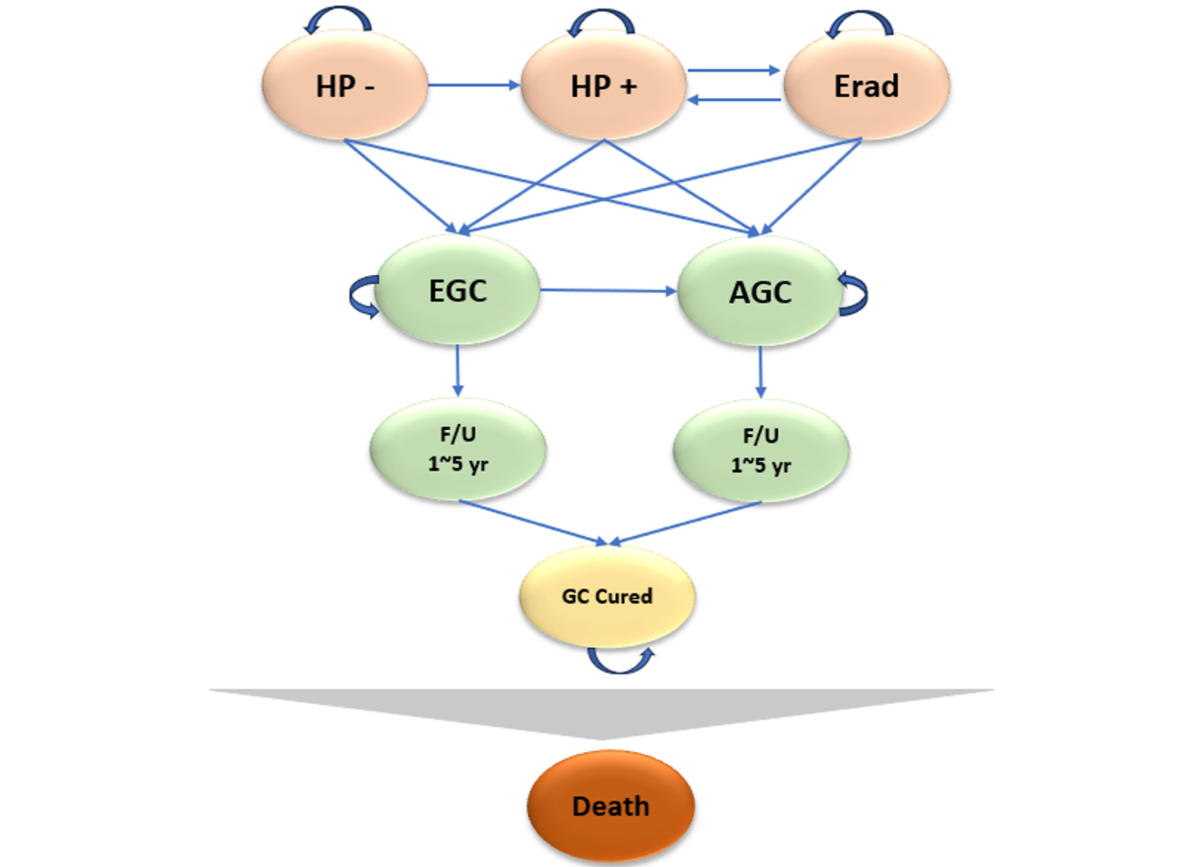
Markov model based on Helicobacter pylori and gastric cancer natural history. AGC: advanced gastric cancer; EGC: early gastric cancer; Erad: eradicated; F/U: follow/up; GC: gastric cancer; HP: Helicobacter pylori.

The Markov simulation model was constructed for a hypothetical population starting at 40 years, with cycles repeated until death or up to a maximum age of 120 years, for a total of 81 cycles; 1 cycle indicates 1 year. The main outcome presents the estimated cumulative costs and quality-adjusted life-years (QALYs) for a person’s entire life cycle. At the beginning of the cohort, individuals are categorized as either noninfected or infected with *H pylori*, and those who successfully undergo eradication therapy transition to the eradicated state. Reinfection is possible even in the eradicated state, and it is assumed that natural eradication does not occur and that infection is only eradicated by treatment. In all states, individuals may transition to either early or advanced gastric cancer. Upon cancer onset, patients remain in a cancer-cured state after a 5-year follow-up period. Figure 1 shows the Markov model. This study aims to verify whether introducing *H pylori* testing and eradication therapy is cost-effective, and to determine whether it reduces the risk of developing early gastric cancer compared to conventional screening.

### Parameters

In this study, the parameters required for the model are categorized as related to epidemiology, cost, or screening. Epidemiological parameters include the prevalence of *H pylori* infection, annual infection rate, reinfection rate, probability of progression to cancer, and mortality rate (among the general population and patients with cancer), which can be obtained from sources such as the National Health Insurance Service (NHIS) database, national statistics, and prior studies [3,13-20]. Cost parameters include the costs associated with *H pylori* testing and eradication therapy (such as biopsy costs and medication costs), the cost of endoscopy screening, and the cost of cancer treatment. Following the economic evaluation guideline of the Health Insurance Review and Assessment Service, we take a health care system perspective, so only direct costs, excluding transportation and caregiving expenses, are considered. Cost data are obtained through the National Health Insurance Medical Service Benefit Costs, the price by main ingredient of pharmaceutical products, and the NCSP guidelines [21]. Finally, the screening parameters include the rate of *H pylori* testing and rate of eradication therapy if the test result is positive, which is assumed to be 100%. The sensitivity and specificity of the *H pylori* testing and eradication success rates are derived from prior studies [22-25]. The endoscopy screening rate, sensitivity, and specificity are obtained from a combined dataset of the Central Cancer Registry Database and the National Cancer Screening Database of the NHIS. A list of these parameters is presented in Table 1.

**Table 1:** Model parameters.

Parameter		Base case	Sensitivity analysis	Reference
**Epidemiological parameters, %**				
	*H pylori* prevalence rate (from age 40-49 years)	41.5	±10	[3]
	Transition rate from *H pylori* negative to *H pylori* positive	0.4	0.3-0.5	[13]
	Transition rate from *H pylori* negative to EGC^a^	0.14	0.11-0.17	[14]
	Transition rate from *H pylori* negative to AGC^b^	0.073	0.06-0.086	[15]
	Risk ratio of cancer incidence w/wo *H pylori* infection	1.69	1.29-2.22	[16]
	Transition rate from “eradicated” to *H pylori* positive	4.3	1.7-6.9	[17]
	Transition rate from EGC to AGC	29.67	±10	[18]
	Annual mortality rate for EGC patients	0.66	0.4-0.99	[19]
	Annual mortality rate for AGC patients	Age 40-59: 12.3; age 60-79: 15.3; age ≥80: 22.1	—^c^	[20]
**Utility weights, %**				
	*H pylori* infected	0.857	SD 0.22	[26]
	EGC patients	0.773	SD 0.26	[26]
	AGC patients	0.589	SD 0.26	[26]
**Examination parameters, %**				
	*H pylori* test sensitivity	90	85-95	[22-24]
	*H pylori* test specificity	98	96-100	[22-24]
	*H pylori* eradication success rate	76	64-91.4	[25]
**Cost parameters, KRW (1 KRW = US $0.00073)**				
	*H pylori* test cost	83,006	±10	Health insurance benefits
	*H pylori* eradication cost	131,490	±10	Health insurance benefits
	Endoscopy screening cost	118,867	—	[21]
	Sedation cost	50,000	38,771-72,500	HIRA^d^ open data

^a^EGC: early gastric cancer.

^b^AGC: advanced gastric cancer.

^c^Not applicable.

^d^HIRA: Health Insurance Review and Assessment.

### *H Pylori* Screening

An individual receives a rapid urea test at the age of 40 years during endoscopy as part of the NCSP. The patient receives eradication therapy if the urease test results are positive. The eradication regimen consists of a clarithromycin-based triple therapy regimen (twice-daily doses of 40 mg esomeprazole, 1 g amoxicillin, and 500 mg clarithromycin) for 14 days.

### Outcomes

The main outcome of this study is the incremental cost-utility ratio (ICUR). This is calculated by dividing the incremental cost between the two strategies by incremental utility, represented by the QALYs accumulated over all cycles for each strategy. The utility weight for each health state is aggregated to determine the QALYs, with utility weights for the general population obtained from the National Health and Nutrition Examination Survey Database and those for *H pylori*–infected individuals and cancer patients obtained from a prior study [26].

### Uncertainty and Scenario Analysis

A probabilistic sensitivity analysis will be performed to test the uncertainty of the cost-effectiveness results. This involves running the Markov simulation 100,000 times to verify the consistency and robustness of the results. Additionally, a one-way sensitivity analysis will be conducted to identify the variables that have the most significant impact on the results, observing how the cost-effectiveness outcome changes when the values of these variables vary within a specific range.

In the base-case analysis, the *H pylori* testing and eradication therapy implementation rates are assumed to be 100%. However, when these rates vary, the cost-effectiveness of different scenarios can be compared. Therefore, the analysis identifies which scenario, with specific values for testing and eradication therapy implementation rates, is the most cost-effective.

### Ethical Considerations

This study will analyze and synthesize previously published data and is therefore exempt from institutional review board approval from the National Cancer Center.

## Results

The primary outcome parameters are the cumulative costs (in KRW) and QALYs for each strategy. A strategy with higher QALYs indicates higher effectiveness; however, if a strategy leads to cost overloads, it cannot be considered cost-effective. The ICUR predicts the cost-effectiveness of a strategy of adding *H pylori* testing and eradication versus the current strategy of endoscopic cancer screening. The ICUR should be within an advanced threshold set to confirm the cost-effectiveness of a certain strategy. Notably, according to the World Health Organization recommendation to evaluate pharmacoeconomics, for an ICUR <1-fold gross domestic product (GDP) per capita, the increased cost is completely worth it and very cost-effective; for an ICUR >1-fold and <3-fold GDP per capita, the increased cost is acceptable and cost-effective. In Korea, a study reported that the average amount of willingness-to-pay for gaining 1 QALY was KRW 30,500,000 (US $22,398) for the overall severity of health conditions (including mild, moderate, and severe) [27]. Therefore, this will be used as the threshold in this study. Costs and utilities will be discounted at an annual rate of 4.5%, with a range of 3% to 6%, for the sensitivity analysis.

In addition, a tornado diagram will be presented to depict the changes in certain variables that influence the results, and a cost-effectiveness acceptability curve will be drawn to confirm the uncertainty of cost-effectiveness with 100,000 simulations. This research has been funded by a Patient-Centered Clinical Research Coordinating Center grant from the Ministry of Health & Welfare, Republic of Korea. Data collection started in June 2024 and was completed in May 2025. The results of this study will be disseminated through publication in a peer-reviewed scientific journal. In addition, the findings will be presented at relevant national and international conferences throughout 2025. Our results will also be shared with government health care directors to facilitate changes in government health policies.

## Discussion

### Principal Findings

This study aims to provide evidence for the cost-effectiveness of integrating *H pylori* screening into the NCSP to reduce gastric cancer risk. We are planning to determine the most cost-effective methods of *H pylori* screening based on age at screening and the frequency of screening. We expect that our study will also identify factors that contribute to improving cost-effectiveness in the National Gastric Cancer Screening Program. The anticipated findings of our study have the potential to significantly impact gastric cancer screening programs. By examining the cost-effectiveness of *H pylori* screening, we aim to highlight the importance of *H pylori* in preventing gastric cancer. This should lead to a paradigm shift in gastric cancer screening programs, from early detection to primary prevention.

There are expected limitations to our study, such as the age at *H pylori* screening. As the purpose of our study is to incorporate *H pylori* screening into the nationwide gastric cancer screening program, we will examine the cost-effectiveness of screening starting from the age of 40 years, which is when the NCSP begins. Future studies are needed to determine whether early screening for *H pylori* is more cost-effective. Stratified screening based on the risk of gastric cancer development will not be examined in our study due to the lack of data on this topic. We plan to examine whether extending the endoscopic screening interval is cost-effective in patients who have received eradication therapy.

Our cost-effectiveness analysis represents a crucial step toward understanding and promoting the most effective method for gastric cancer screening. We expect to provide evidence for the effectiveness of *H pylori* screening for gastric cancer prevention. The results of our study could serve as a valuable reference for the development of gastric cancer screening programs in other countries.

### Conclusion

We plan to examine whether introducing *H pylori* testing and eradication therapy into the NCSP is a more cost-effective strategy than conventional endoscopy-based cancer screening for reducing the risk of gastric cancer. Our study will also examine the optimal age for *H pylori* screening as well as the optimal screening frequency for *H pylori*.

## Appendix

Multimedia Appendix 1 CHEERS checklist.

## Abbreviations

CHEERS: Consolidated Health Economic Evaluation Reporting Standard
ICUR: incremental cost-utility ratio
NCSP: National Cancer Screening Program
NHIS: National Health Insurance Service
QALY: quality-adjusted life-year

## Data Availability

This study will analyze and synthesize previously published information. Data sharing does not apply to this study because no datasets will be generated or analyzed. The data and references included in the development of the Markov model are presented in Table 1.
